# Supplementary material for: A Gene Co-Expression Network-Based Drug Repositioning Approach Identifies Candidates for Treatment of Hepatocellular Carcinoma
Source: Cancers (Basel). 2022 Mar 19;14(6):1573. doi: 10.3390/cancers14061573 (PMC8946504; doi:10.3390/cancers14061573)
Supplement: Supplementary file 1 [file cancers-14-01573-s001.zip › supplementary Figures.pdf]

Supplementary Figure 1.

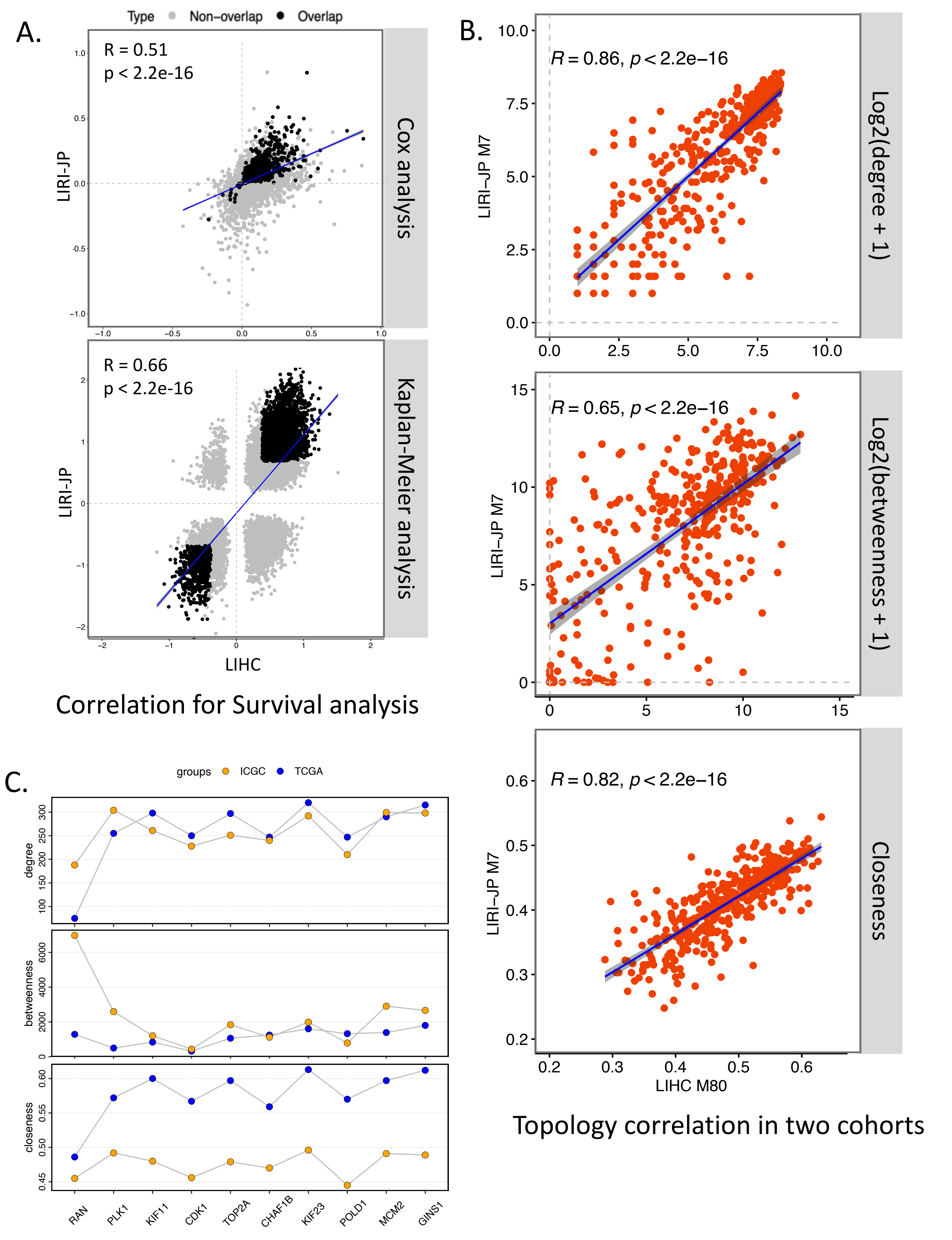

Supplementary Figure 2. Kaplan Meier plots of target ge

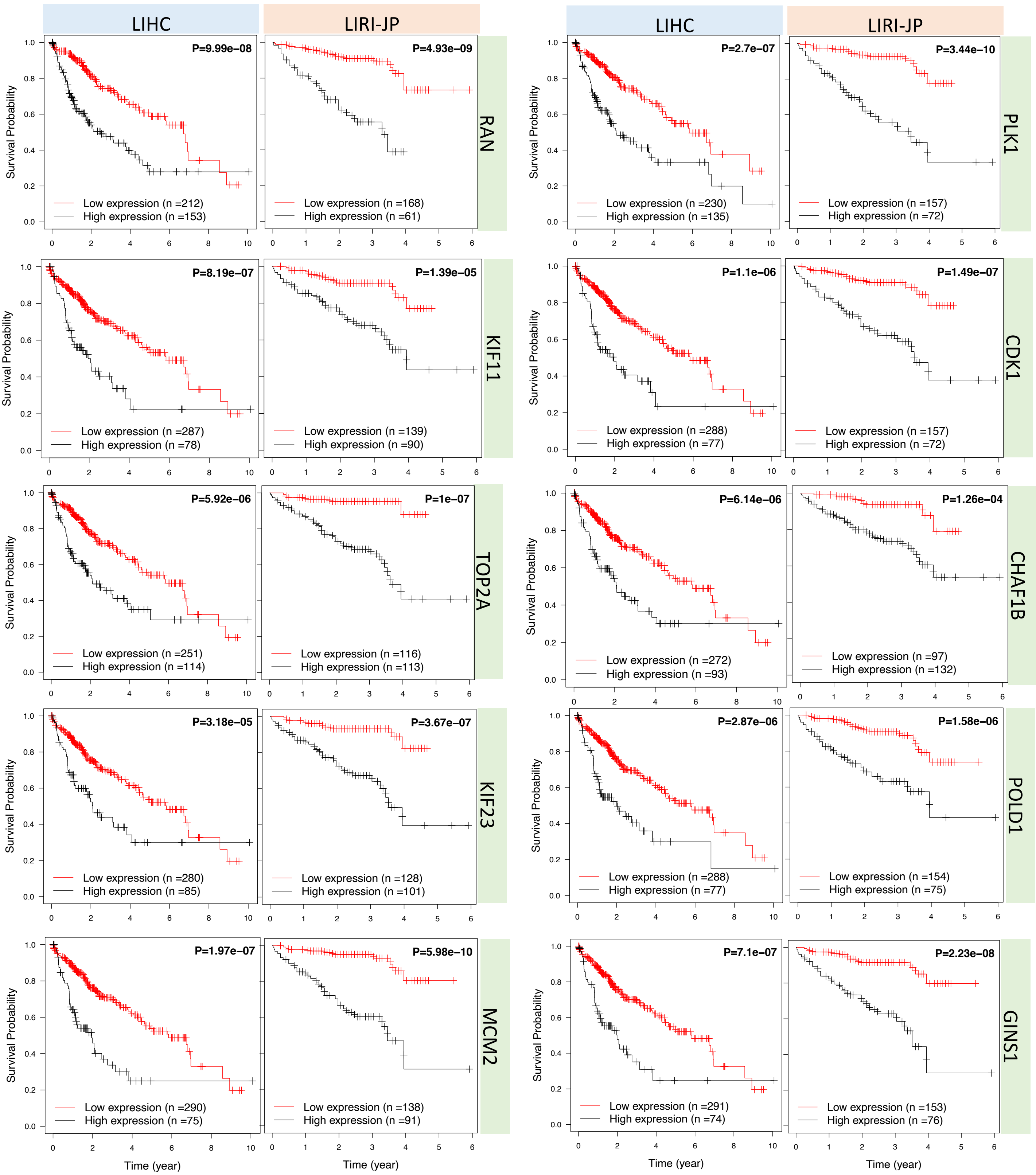

Supplementary Figure 3.
Top drug identification of *KIF11*, *CDK1*, *RAN*, *CHAF1B*.

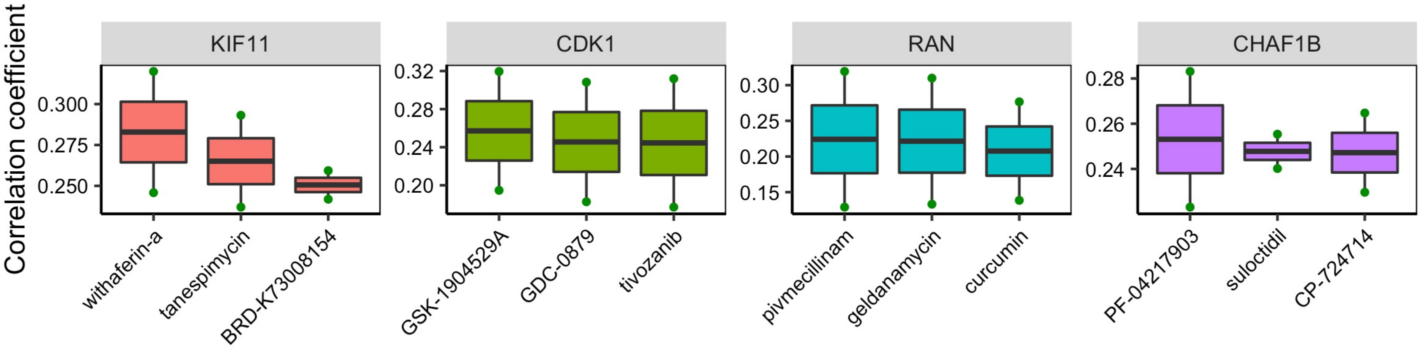

Supplementary Figure 4.

The expression pattern of potential target genes in different cell types

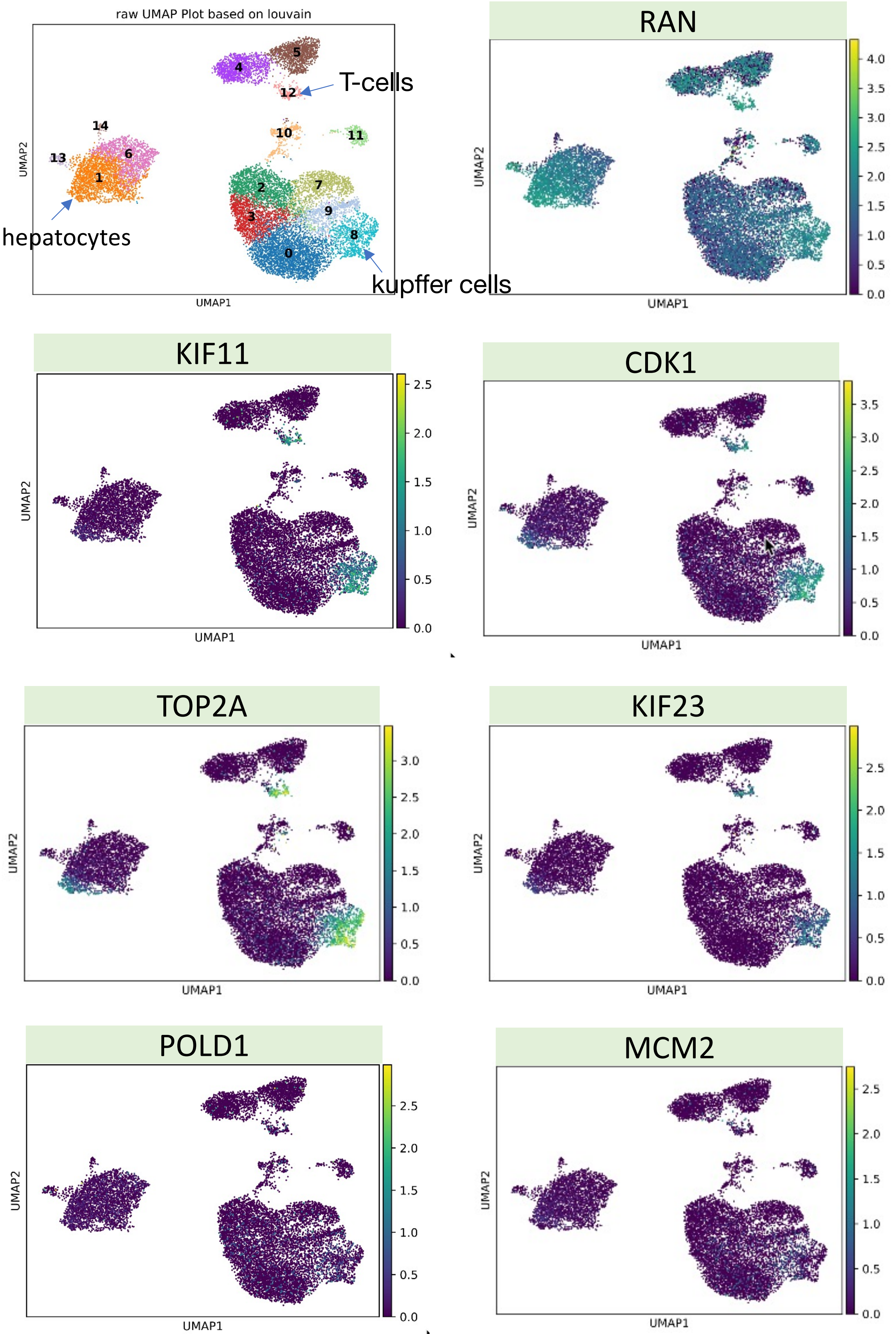

Supplementary Figure 5.
Clustering analysis of shRNAs for target genes

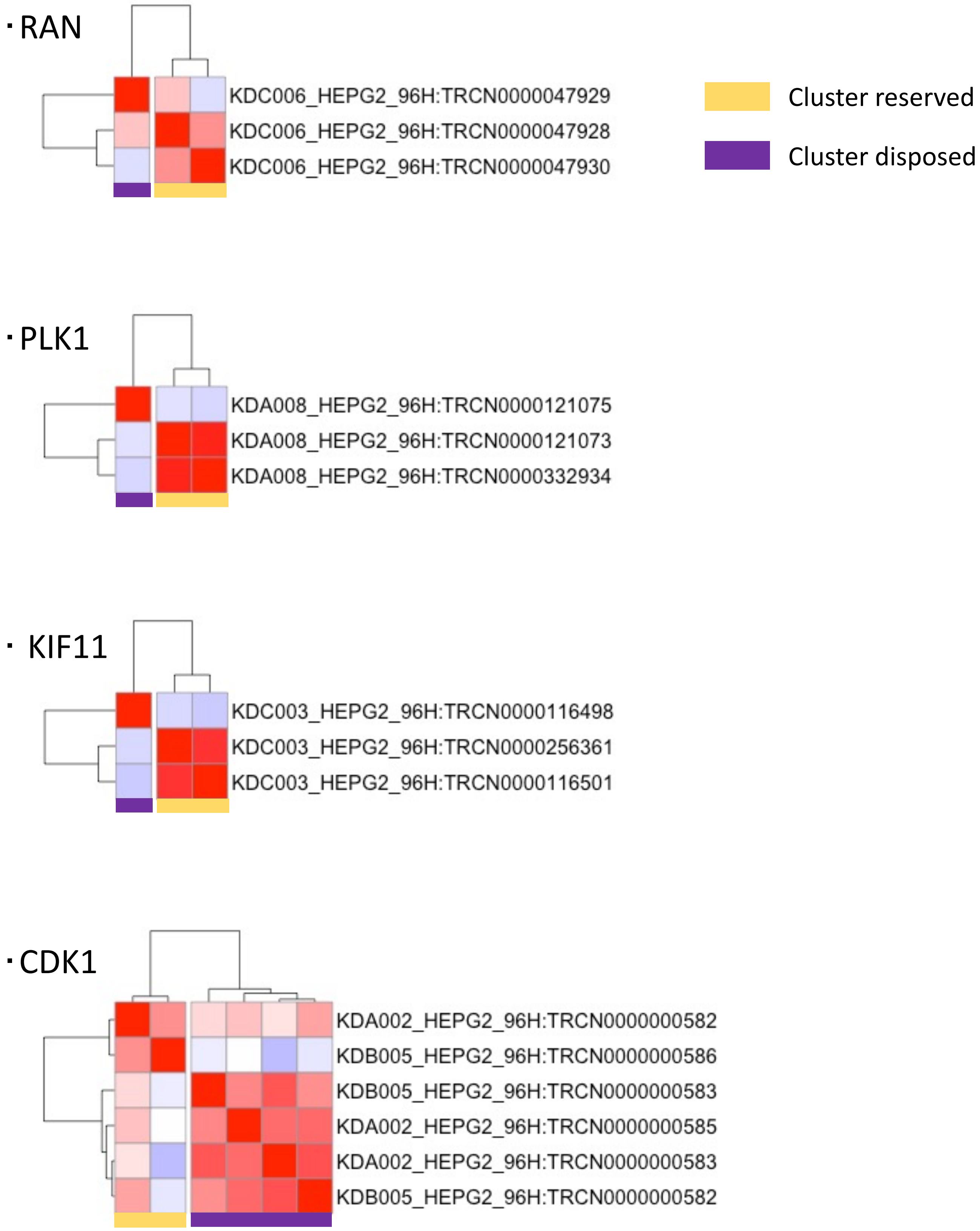

· TOP2A

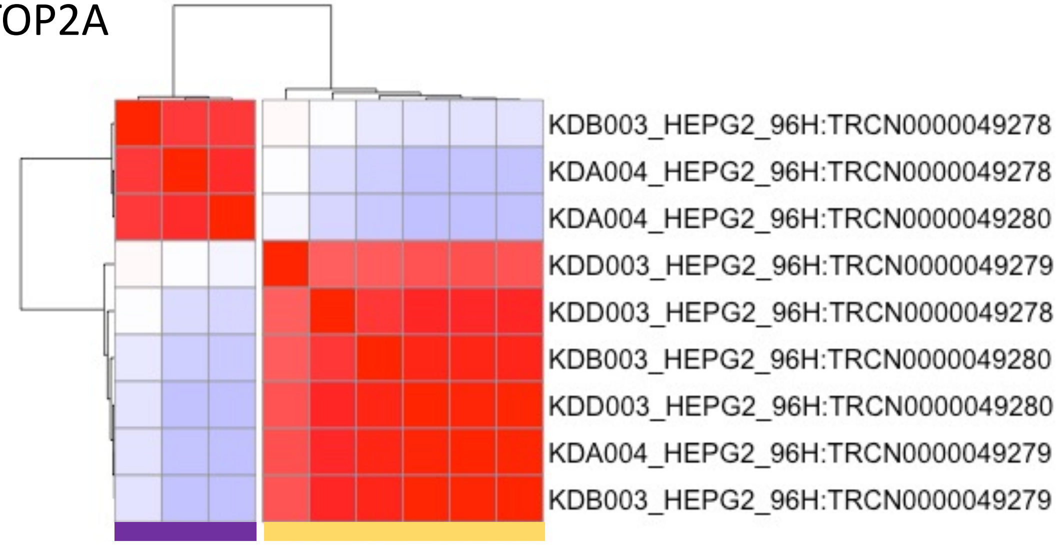

· CHAF1B

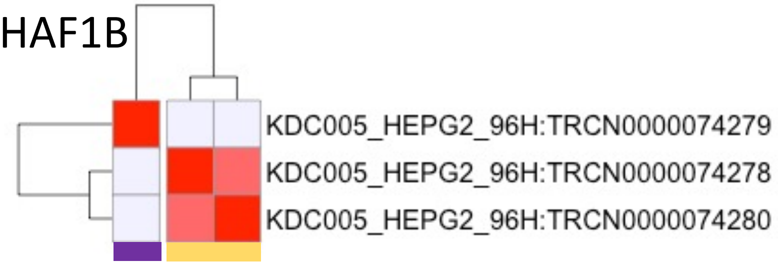

· MCM2

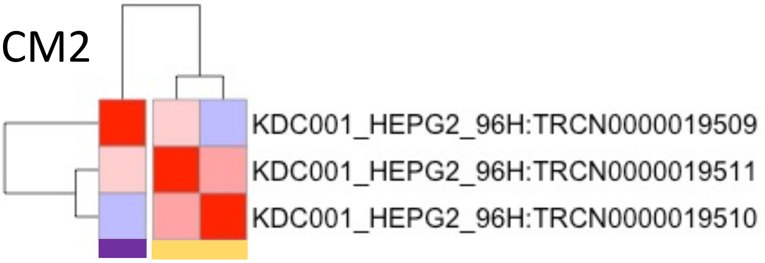

Supplementary Figure 6.
Protein expression levels in in-vitro model

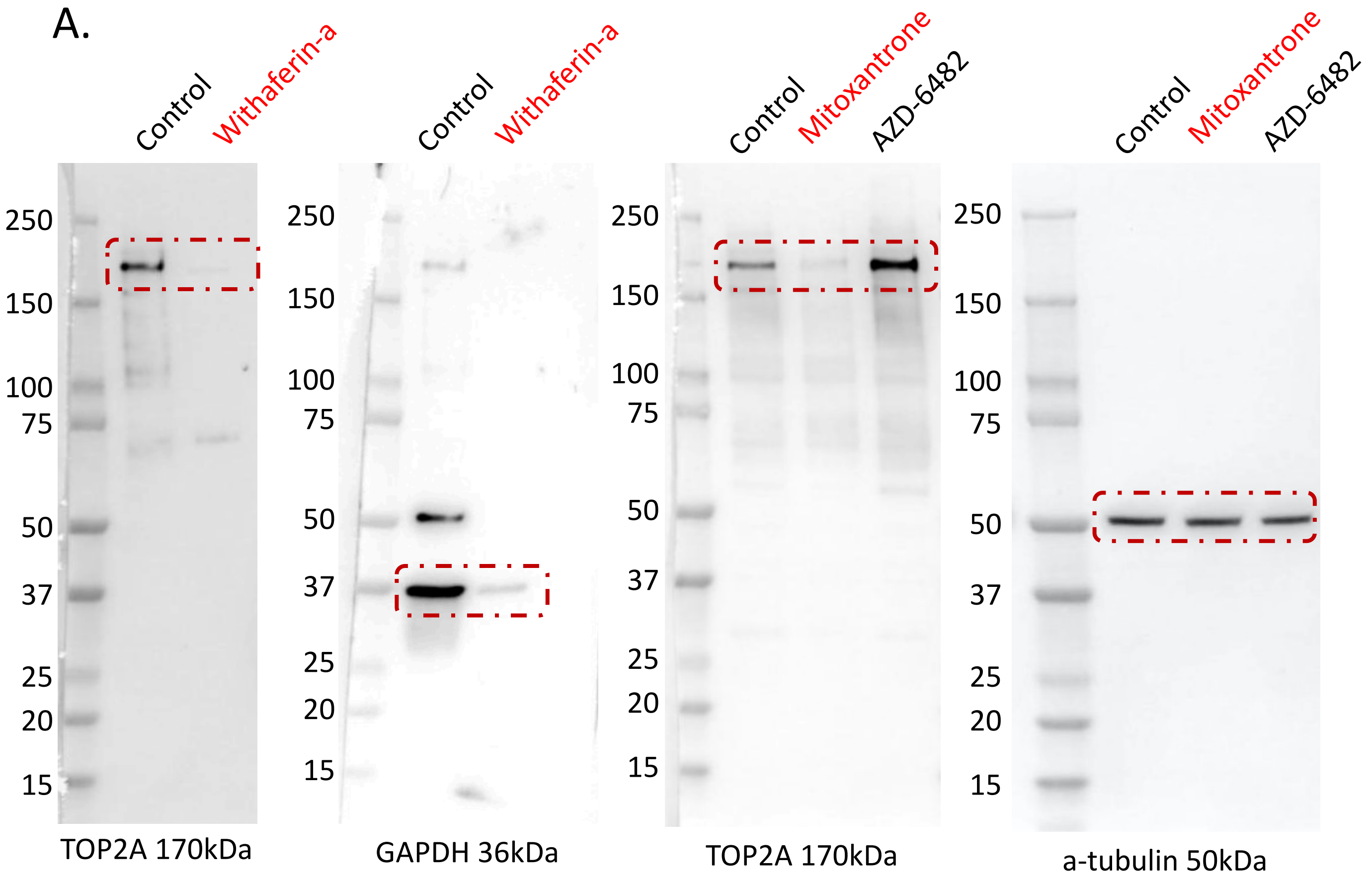

TOP2A (GAPDH)	Intensity ratio
Control	1
Withaferin-a	0.32

TOP2A (A-tubulin)	Intensity ratio
Control	1
Mitoxantrone	0.43
AZD-6482	2.57

B.

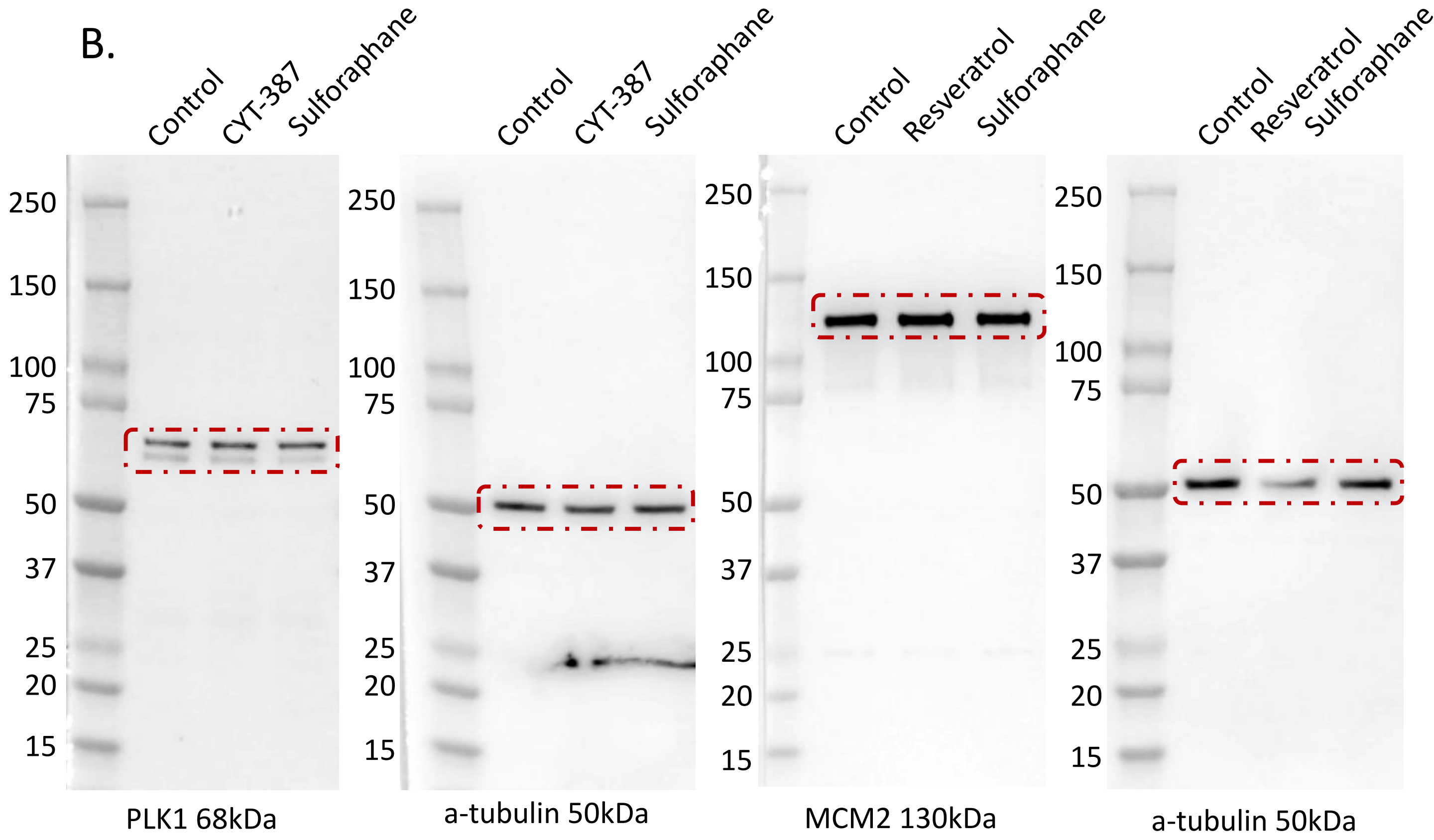

PLK1	Intensity ratio
Control	1
CYT-387	1.21
Sulforaphane	0.98

MCM2	Intensity ratio
Control	1
Resveratrol	2.44
Sulforaphane	1.13
